# Factors associated with quality of life in patients with Alzheimer’s disease

**DOI:** 10.1186/s12877-018-0855-7

**Published:** 2018-07-09

**Authors:** Coralie Barbe, Damien Jolly, Isabella Morrone, Aurore Wolak-Thierry, Moustapha Dramé, Jean-Luc Novella, Rachid Mahmoudi

**Affiliations:** 1 Faculty of Medicine, EA 3797, University of Reims Champagne-Ardenne, F-51092 Reims, France; 2 Department of Research and Public health, Reims University Hospitals, Robert Debré Hospital, F-51092 Reims, France; 3 Department of Geriatrics and Internal Medicine, Reims University Hospitals, Maison Blanche Hospital, F-51092 Reims, France

**Keywords:** Alzheimer’s disease, Quality of life, QoL-AD, Associated factors

## Abstract

**Background:**

Evaluation of health-related quality of life (HRQoL) in patients with Alzheimer’s disease (AD) is necessary to ensure optimal management. Several scales for assessing HRQoL of patients with AD exist, in particular the Quality of Life in Alzheimer’s Disease (QoL-AD), which includes an evaluation by the caregiver of the patient’s HRQoL. The aim of this study was to identify factors associated with patient, caregiver and overall HRQoL as assessed by the QoL-AD.

**Methods:**

Cross-sectional multicenter study in subjects aged 65 years and older, with mild to moderate AD. HRQoL scores from the QoL-AD were recorded (3 scores, corresponding to patient, caregiver and overall), as well as sociodemographic variables for the patient and the caregiver, and data from the geriatric cognitive assessment (cognitive, psycho-behavioral, functional evaluations). Caregiver burden was evaluated using the Zarit caregiver burden scale. Factors associated with each QoL-AD score were identified by multivariate linear regression using t-tests and β estimations. Study was registered in Clinical Trial.gov (NCT02814773).

**Results:**

In total, 123 patients with AD were included. For the patient QoL-AD evaluation, depression was significantly associated with lower HRQoL (β = − 2.56 ± 1.28, *p* = 0.04), while polypharmacy (β = − 1.80 ± 0.99, *p* = 0.07) and anxiety (β = − 1.70 ± 1.01, *p* = 0.09) tended to be associated with lower HRQoL scores. In terms of caregiver evaluations, depression (β = − 3.46 ± 1.09, *p* = 0.002), polypharmacy (β = − 1.91 ± 0.92, *p* = 0.04) and the presence of caregiver burden (β = − 3.50 ± 0.91, *p* = 0.0002) were associated with lower HRQoL. For the overall evaluation, depression (β = − 3.26 ± 1.02, *p* = 0.002) and polypharmacy (β = − 1.85 ± 0.81, *p* = 0.03) were significantly related to lower HRQoL.

**Conclusions:**

Depression and polypharmacy were two factors influencing HRQoL in patients with AD, both by patient self-report and on the caregiver report. Thus, despite the discrepancies between HRQoL as assessed by patients with AD and HRQoL as assessed by their caregiver, the caregiver’s assessment may be used to guide patient management when the patient can no longer complete QoL evaluations. Moreover, the association between caregiver burden and the caregiver’s QoL-AD score underlines the need to take caregivers into consideration in the overall management of the AD patient.

## Background

Alzheimer’s disease (AD) is a chronic and progressive neurodegenerative disorder that affects cognitive functions such as memory, behavior and affective capacity, and leads to progressive loss of autonomy and the ability to partake in social activities (https://www.alz.co.uk/research/files/WorldAlzheimerReport.pdf). AD affects the daily lives not only of the patients but also of their entourage. Management of patients with AD is based on a multidisciplinary approach (medical, cognitive, psychological, social and functional), with the primary aim of maintaining the patient’s well-being, and more generally, their quality of life (QoL) [1]. Cognitive, psycho-behavioural and functional assessment makes it possible to evaluate the impact of the disease on the overall capacity of a patient with AD. However, it does not inform about the patient’s own perception of how the disease affects them. Yet, the patient’s own point of view is a key element in developing a tailored healthcare plan.

Health-related quality of life (HRQoL) is a vast concept that encompasses physical and mental health, autonomy, social interactions and the relationship between a subject and their environment. Evaluating HRQoL in patients with AD is necessary to ensure optimal management, as it reflects the patient’s own perception of the impact of their disease. Several specific scales for measuring HRQoL in patients with dementia have been developed [2]. HRQoL assessment is highly subjective, since it reflects patients’ personal experiences [1]. Nonetheless, although self-assessment instruments can be used at the early stages of the disease, the progression of cognitive decline makes their use at later stages more difficult. Furthermore, the typology of cognitive impairment, the presence or absence of anasognosia, and the inevitable progression of the disease, make evaluation by a family member, relative or other loved one an attractive solution. Accordingly, certain HRQoL instruments comprise both self-reports and caregiver reports, such as the Quality of Life in Alzheimer Disease (QoL-AD) instrument, which comprises both patient and caregiver reports of patient HRQoL [3]. Yet, evaluation of the patient’s HRQoL by a third party is debatable, and numerous studies have shown discrepancies between HRQoL as assessed by patients with AD, and the corresponding evaluation by their caregiver, mostly observing that loved ones underestimate the patient’s HRQoL [4–8].

The aim of this study was to identify factors associated with HRQoL in patients with AD, as assessed by the patient score, the caregiver score and the overall score on the QoL-AD. Relations between the determinants of these 3 scores and the 13 individual items of the QoL-AD were also analysed.

## Methods

### Patient inclusions

A multicentre, cross-sectional study in 7 French-speaking hospitals (Reims, Paris, Nancy, Dijon, Orléans, Joeuf, (France) and Geneva (Switzerland)) was performed between 2006 and 2008. Patients were recruited in memory clinics or geriatric medicine wards. To be eligible for inclusion, patients had to be aged 65 years or older, with mild to moderate Alzheimer-type dementia according to the Diagnostic and Statistical Manual of Mental Disorders, 4th Edition, Text Revision (DSM-IV-TR) [9] and the National Institute of Neurological and Communicative Disorders and Stroke (NINCDS) and the Alzheimer’s Disease and Related Disorders Association (ADRDA) criteria [10]. They also had to have a primary caregiver. Patients protected by law were not included.

The Institutional Review Board of the University Hospital of Reims, France, approved the study protocol. This non-interventional study did not in any way change routine care of the patients included. A double informed consent form was signed, by both the patient and their main caregiver. According to French law, patients could withdraw from the study at any time. Withdrawal from the study in no way affected subsequent management.

### Data recorded

Data were recorded by a geriatrician during physical examination of the patient, and during the interview with the patient and caregiver.

Patient HRQoL was measured using the QoL-AD, which is a tool that is already validated in both English [3] and French [11]. This questionnaire comprises 13 items addressing the following points: physical health, energy levels, mood, living situation, memory, relationship with friends, relationship with family members, relationship with spouse, self-esteem, ability to do chores around the house, ability to do things for fun, financial situation and life as a whole. Each item is scored on an ordinal scale from 1 (poor) to 4 (excellent). The questionnaire is completed by the patient and by the caregiver (or informant) for the patient. Three QoL scores ranging from 13 to 52 are thus obtained, namely the patient score, the caregiver score and the overall score. The overall score is calculated as: [(patient score × 2) + caregiver score]/3 [3]. The higher the score, the better the HRQoL.

Functional capacities of the subjects was evaluated using 2 instruments, namely Katz’s 6-item Activities of Daily Living (ADL) (i.e. bathing, dressing, toileting, transferring, continence, feeding) [12] and Lawton’s Instrumental Activities of Daily Living (IADL) as modified by the PAQUID study to comprise 4 items, namely ability to use the telephone, do the shopping, use public transport and manage own medication [13]. For the ADL, a subject was considered independent if they could carry out the tasks without help. For the continence item of the ADL, the subject was considered not to be continent if there was occasional or complete incontinence.

Sociodemographic data pertaining to the patient (age, sex, level of education) and the caregiver (age, sex, relation to the patient) were also recorded. The presence of formal help (home nurse, home help, delivery of meals, attendance at a day-care centre) was evaluating using the Resource Utilization in Dementia (RUD) instrument [14]. Existence of caregiver burden was evaluated using the Zarit caregiver burden scale [15], with a score of 21 or more indicating the presence of caregiver burden.

Clinical evaluation of patients was performed at inclusion. The Mini Mental State Examination (MMSE) score was recorded at admission [16]. Diagnoses were coded using the International Classification of Diseases version 10 (ICD10). The Charlson index was used to assess comorbidities [17]. We noted whether the patient was taking symptomatic treatment for AD (anti-cholinesterase drugs or glutamate receptors), as well as the use of psychotropic agents. The total number of drugs being taken by the patient was also noted, and polypharmacy was defined as 3 or more drugs per day [18]. Nutritional status was assessed by calculating body mass index (BMI). Obesity was defined as a BMI > 30 kg/m^2^ and malnutrition as a BMI < 21 kg/m^2^) [19].

The Neuro-Psychiatric-Inventory (NPI) [20] was used to assess the presence of psycho-behavioural disorders. The NPI comprises 12 items. The overall score is the sum of the severity (maximum 36 points) and distress (maximum 60 points) scores, and a higher score reflects more serious symptoms and/or greater distress for the caregiver. In addition to the overall score, the specific presence of each behavioural disorder was studied.

The Cornell scale for depression in dementia [21] was used to investigate the presence of depression in patients, with a score of 10 or more indicating the presence of depression.

### Statistical analysis

Quantitative variables are reported by their mean ± standard deviation and qualitative variables by number (percentage). The factors associated with each of the three scores on the QoL-AD were investigated by bivariate analysis (Student t, Wilcoxon rank tests or Pearson’s correlation coefficient, as appropriate). Multiple regressions (backward linear regression, with an exit threshold of 0.10, β estimations and tests to zero by t-test) were performed with scores on the QoL-AD as dependent variables. For each multivariate model, all variables with a *p*-value < 0.10 by univariate analysis were included. The conditions of validity of the linear regression models (normality of residuals, homoscedasticity and independence of residuals) were verified using graphical methods. The associations between the component items of the QoL-AD (qualitative ordinal variables) and the factors identified by multiple regression to be related to the patient, caregiver, or overall QoL-AD scores were investigated using the Cochran Armitage trend test. A two-sided *p*-value < 0.05 was considered as statistically significant. All analyses were performed using SAS version 9.4 (SAS Institute Inc., Cary, NC, USA).

## Results

### Study population

In total 123 patients with AD were included. The baseline characteristics of the study population are shown in Table 1. Average age was 82.0 ± 6.2 years; the majority (63.4%) were women. Mean MMSE score was 20.7 ± 4.5, and 50.4% of patients had mild AD. One third (33.1%) had attended school up to the level of a high-school diploma. Caregivers were informal caregivers in 110 patients (82.1%), namely the spouse in 66 (65.3%), a child in 28 (27.7%) and other family in 7 patients (6.9%). Formal caregivers were a home nurse in 10 patients (45.5%) and a nursing auxiliary in 12 patients (54.5%). At inclusion, the average HRQoL scores as measured by the QoL-AD were 36.1 ± 5.0 for the patient score, 32.7 ± 4.9 for the caregiver score, and 35.0 ± 4.3 for the overall score. No participant refused administration of the QoL-AD, no participant dropped out during the course of administration. Four patients and nine caregivers had one or two non-completed items. These missing data were imputed using the procedure recommended by Logsdon (substitution by the mean score of the other items) [3].

**Table 1:** Baseline characteristics of the study population of 123 subjects with Alzheimer’s Disease

Variables^a^	Patients *N* = 123
BMI^b, c^	24.9 ± 4.1
Malnourished (BMI < 21 kg/m^2^)^c^	18 (15.4)
Charlson comorbidity index^b, d^	1.35 ± 1.11
Number of medications per day^b, e^	4.2 ± 2.6
Polypharmacy (> 3 medications per day) ^e^	61 (51.3)
Psychotropic drugs^d^	69 (57.5)
Symptomatic treatment of AD ^f^	106 (87.6)
Depression ^c^	20 (19.6)
ADLs ^b, g^	5.1 ± 1.2
Washing ^g^	77 (63.1)
Dressing ^g^	83 (68.0)
Toileting ^g^	106 (86.9)
Transferring ^g^	100 (82.0)
Continence ^g^	79 (64.7)
Feeding ^g^	110 (90.2)
IADL (not sex dependent) ^b, e^	1.8 ± 1.2
Ability to use telephone ^g^	105 (86.1)
Ability to do shopping ^e^	25 (20.8)
Ability to use transport ^g^	61 (50)
Ability to manage own treatment ^f^	29 (24.0)
Caregiver’s age ^b, d^	66.3 ± 14.3
Caregiver’s sex	
Male	36 (29.3)
Female	87 (70.7)
Relationship to caregiver: spouse / child	94 (76.4)
Home nurse ^h^	18 (17.1)
Home help for housework ^i^	57 (53.8)
Home delivery of meals ^i^	9 (8.5)
Attending daycare center ^j^	21 (20.2)
NPI score ^b, d^	13.0 ± 11.7
Delusions ^g^	24 (19.7)
Hallucinations ^d^	8 (6.7)
Agitation/aggression ^g^	54 (44.3)
Dysphoria/depression ^k^	6 (5.6)
Anxiety ^g^	76 (62.3)
Eurphoria ^g^	34 (27.9)
Apathy ^g^	65 (53.3)
Disinhibition ^g^	25 (20.5)
Aberrant motor behaviour ^e^	9 (7.5)
Irritability ^g^	62 (50.8)
Sleep & nighttime behaviour change ^g^	39 (32.0)
Appetite & eating change ^g^	33 (27.0)
Caregiver burden ^l^	62 (60.8)

^a^n (%) unless otherwise indicated, ^b^ mean ± standard deviation. ^c^ 6 missing data, ^d^ 3 missing data, ^e^ 4 missing data, ^f^ 2 missing data, ^g^ 1 missing data, ^h^18 missing data, ^i^ 17 missing data; ^j^ 19 missing data, ^k^ 15 missing data, ^l^21 missing data

*MMSE* Mini Mental State Examination, *BMI* body mass index, *AD* Alzheimer’s disease, *ADLs* Activities of Daily Living, *IADL* Instrumental Activities of Daily Living, *NPI* NeuroPsychiatric Inventory

### QoL-AD patient score

By bivariate analysis, the factors shown to be associated with the QoL-AD patient score were patient age (*r* = − 0.17; *p* = 0.05), polypharmacy (35.0 ± 4.7 versus 37.0 ± 5.2; *p* = 0.03), symptomatic treatment for AD (36.6 ± 4.8 versus 33.9 ± 5.1; *p* = 0.03), depression as assessed by Cornell’s scale (33.3 ± 4.6 versus 37.1 ± 5.0; *p* = 0.005) and anxiety as assessed by the NPI (35.4 ± 5.0 versus 37.3 ± 4.9; *p* = 0.04). Variables included in the multivariate analysis were patient age, polypharmacy, use of psychotropic agents, symptomatic treatment for AD, depression (Cornell’s scale), preserved motor capacity (ADL) and anxiety (NPI). In the multiple regression (Table 2), the presence of depression as assessed by Cornell’s scale was significantly associated with lower QoL score (β = − 2.56 ± 1.28, *p* = 0.04). Polypharmacy (β = − 1.80 ± 0.99, *p* = 0.07) and anxiety, as assessed by the NPI, (β = − 1.70 ± 1.01, *p* = 0.09) tended to be associated with lower QoL score, albeit without reaching statistical significance. Analysing the items of the QoL-AD individually (Table 3), a significant decrease in the depression rate across the range of responses (i.e. from item response 1 (poor HRQoL) to item response 4 (excellent HRQoL)) was shown for the following items: mood (*p* = 0.0002), ability to do things for fun (*p* = 0.01), life as a whole (*p* = 0.0009) and memory (*p* = 0.02). A significant decrease in the rate of polypharmacy from item response 1 (poor HRQoL) to item response 4 (excellent HRQoL) was observed for the physical health item (*p* < 0.0001), and a significant decrease in the rate of anxiety from item response 1 (poor HRQoL) to item response 4 (excellent HRQoL) was observed for the following items: mood (*p* = 0.03), memory (*p* = 0.001) and self-esteem (*p* = 0.04).

**Table 2:** Multivariate analysis investigating determinants of the patient, caregiver and overall scores on the QoL-AD

Variables	Patient score (*n* = 99)			Caregiver score (*n* = 84)			Overall score (*n* = 100)		
	QoL-AD score^a^	Multivariate analyse^b^		QoL-AD score^a^	Multivariate analyse^c^		QoL-AD score^a^	Multivariate analyse^d^	
		β ± SE (β)	p		β ± SE (β)	p		β ± SE (β)	p
Depression			**			***			***
yes	33.3 ± 4.6	−2.56 ± 1.28		29.8 ± 3.8	−3.46 ± 1.09		32.1 ± 3.6	−3.26 ± 1.02	
no	37.1 ± 5.0			33.7 ± 4.5			36.0 ± 4.1		
Polypharmacy			*			**			**
yes	35.0 ± 4.7	−1.80 ± 0.99		31.6 ± 4.5	−1.91 ± 0.92		33.9 ± 4.0	−1.85 ± 0.81	
no	37.0 ± 5.2			33.7 ± 5.1			35.9 ± 4.4		
Anxiety (NPI)			*						
yes	35.4 ± 5.0	−1.70 ± 1.01							
no	37.3 ± 4.9								
Carer burden						***			
yes				31.0 ± 4.8	−3.50 ± 0.91				
no				35.3 ± 4.1					

*p < 0.10; **p < 0.05; ***p < 0.001

Polypharmacy: > 3 medications per day;

*NPI* NeuroPsychiatric Inventory

^a^mean ± standard deviation

^b^variables included in the multivariate analysis were patient’s age, polypharmacy, use of psychotropic agents, symptomatic treatment for AD, depression (Cornell’s scale), preserved motor capacity (ADL) and anxiety (NPI)

^c^variables included in the multivariate analysis were polypharmacy, depression (Cornell’s scale), presence of home nurse, agitation/aggression (NPI), apathy (NPI), disinhibition (NPI), aberrant motor behaviour (NPI), irritability (NPI), sleep and nighttime behaviour change (NPI), appetite & eating change (NPI) preserved motor capacity (ADL), ability to do shopping (IADL), ability to use transport (IADL) and existence of caregiver burden

^d^variables included in the multivariate analysis were patient’s age, polypharmacy, use of psychotropic agents, symptomatic treatment for AD, depression (Cornell’s scale), agitation/aggression (NPI), anxiety (NPI), sleep and nighttime behaviour change (NPI), preserved motor capacity (ADL) and existence of caregiver burden

**Table 3:** Associations between the factors influencing patient HRQoL and the 13 items of the QoL-AD

	Patient			Caregiver		
	Depression	Polypharmacy	Anxiety (NPI)	Depression	Polypharmacy	Caregiver burden
Physical health		*		*	*	
Energy				*		*
Mood	*		*	*	*	*
Living situation						
Memory	*		*			*
Family						*
Marriage						*
Friends						*
Self as a whole			*	*		
Ability to do chores						*
Ability to do things for fun	*			*	*	*
Financial situation						
Life as a whole	*			*		*

*p < 0.05 (Cochrane Armitage test for trend). For all significant tests, a decrease in the rate of the associated factor (depression, polypharmacy, anxiety or caregiver burden) was observed across the spectrum of responses of the QoL-AD (i.e. from response 1 (poor HRQoL) to item response 4 (excellent HRQoL))

For example, for the item “mood” of the patient QoL-AD, the proportion of patients with depression was 67% for response “1”, 29% for response “2”, 10% for response “3” and 0% for response “4” (*p* = 0.0002)

Polypharmacy: > 3 medications per day;

*NPI* NeuroPsychiatric Inventory

### QoL-AD caregiver score

By bivariate analysis, the factors shown to be associated with the caregiver scores on the QoL-AD were polypharmacy (31.6 ± 4.5 versus 33.7 ± 5.1; *p* = 0.02), depression as assessed by Cornell’s scale (29.8 ± 3.8 versus 33.7 ± 4.5; *p* = 0.0004), preserved motor capacity as evaluated by the ADLs (33.2 ± 4.9 versus 30.8 ± 4.5; *p* = 0.04), agitation/aggression as assessed by the NPI (31.6 ± 4.7 versus 33.6 ± 4.9; *p* = 0.02), apathy as assessed by the NPI (31.7 ± 5.0 versus 33.9 ± 4.5; *p* = 0.01), disinhibition as assessed by the NPI (31.0 ± 5.0 versus 33.2 ± 4.4; *p* = 0.04), irritability as assessed by the NPI (31.7 ± 5.0 versus 33.8 ± 4.5; *p* = 0.01), sleep and nighttime behaviour change as assessed by the NPI (30.4 ± 5.3 versus 33.0 ± 4.7; *p* = 0.0003) and existence of caregiver burden (31.0 ± 4.8 versus 35.3 ± 4.1; *p* < 0.0001). Variables included in the multivariate analysis were polypharmacy, depression (Cornell’s scale), presence of home nurse, agitation/aggression (NPI), apathy (NPI), disinhibition (NPI), aberrant motor behaviour (NPI), irritability (NPI), sleep and nighttime behaviour change (NPI), appetite & eating change (NPI) preserved motor capacity (ADL), ability to do shopping (IADL), ability to use transport (IADL) and existence of caregiver burden. In the multiple regression (Table 2), the existence of depression as measured by Cornell’s scale (β = − 3.46 ± 1.09, *p* = 0.002), polypharmacy (β = − 1.91 ± 0.92, *p* = 0.04) and the presence of caregiver burden (β = − 3.50 ± 0.91, *p* = 0.0002) were significantly associated with lower QoL score. Analysing of the individual items of the QoL-AD (Table 3), a significant decrease in the rate of depression rate from item response 1 (poor HRQoL) to item response 4 (excellent HRQoL) was shown for the following items: mood (*p* < 0.0001), ability to do things for fun (*p* = 0.004), life as a whole (*p* = 0.0004), physical health (*p* = 0.02), energy levels (*p* = 0.01) and self-esteem (*p* = 0.02). A significant decrease in the rate of polypharmacy from item response 1 (poor HRQoL) to item response 4 (excellent HRQoL) was observed for the following items: physical health (*p* = 0.003), mood (*p* = 0.03) and ability to do things for fun (*p* = 0.005). A significant decrease of in the rate of caregiver burden from item response 1 (poor HRQoL) through item response 4 (excellent HRQoL) was shown for the following items: energy levels (*p* = 0.005), mood (*p* = 0.05), memory (*p* = 0.006), relationship with family (*p* = 0.04), relationship with spouse (*p* = 0.009), relationship with friends (*p* = 0.001), ability to do chores (*p* = 0.01), ability to do things for fun (*p* = 0.0002) and life as a whole (*p* = 0.01).

### QoL-AD overall score

By bivariate analysis, the factors shown to be associated with the overall QoL-AD score were polypharmacy (33.9 ± 4.0 versus 35.9 ± 4.4; *p* = 0.008), depression as assessed by Cornell’s scale (32.1 ± 3.6 versus 36.0 ± 4.1; *p* = 0.0007), preserved motor capacity as evaluated by the ADLs (35.5 ± 4.0 versus 32.9 ± 4.7; *p* = 0.02), anxiety as assessed by the NPI (34.3 ± 4.4 versus 36.1 ± 3.9; *p* = 0.03), sleep and nighttime behaviour change as assessed by the NPI (33.7 ± 3.8 versus 35.6 ± 4.4; *p* = 0.02) and existence of caregiver burden (34.5 ± 4.1 versus 36.2 ± 3.8; *p* = 0.04). Variables included in the multivariate analysis were patient age, polypharmacy, use of psychotropic agents, symptomatic treatment for AD, depression (Cornell’s scale), agitation/aggression (NPI), anxiety (NPI), sleep and nighttime behaviour change (NPI), preserved motor capacity (ADL) and existence of caregiver burden. In the multiple regression (Table 2), the presence of depression (β = − 3.26 ± 1.02, *p* = 0.002) and polypharmacy (β = − 1.85 ± 0.81, *p* = 0.03) were found to be significantly related to lower QoL score.

## Discussion

Our study evaluated the factors associated with the different QoL scores measured by the QoL-AD instrument, namely the patient score, the caregiver (informant) score, and the overall score. In our study, depression and polypharmacy were found to be associated with the patient score on the QoL-AD, but also with the caregiver score.

Depression is a frequent comorbidity in patients with AD [22]. A link between depression and both the patient and caregiver scores of the QoL-AD has previously been reported by Chan et al. in a study of 111 patients with AD [23], with poorer QoL observed in patients with depression. Several other studies have reported an association between the presence of depression and impaired QoL in AD patients, regardless of whether the QoL was self-reported or evaluated by others [3, 5, 7, 24]. Depression was the factor that most strongly influenced QoL as evaluated by the patient, with the highest β coefficient (compared to polypharmacy and anxiety). Regarding the caregiver’s evaluation, depression was also found to strongly influence QoL, with a β coefficient of the same magnitude as that of caregiver burden. The findings of our study therefore underscore the importance of identifying and treating depressive symptoms in AD patients. However, detecting depression can be difficult, since certain depressive symptom may be mistaken for symptoms of dementia, such as apathy or decreased energy [25]. Special attention should therefore be paid to these types of symptoms both by healthcare professionals and the patient’s close entourage. Antidepressants may treat depression in older adults but they incur a risk of adverse events because of comorbidities and drug-drug interactions in case of polypharmacy. For these reasons, older people receive lower-than-recommended doses of antidepressants, or are treated for too brief a period. Nonpharmacological interventions such as psychotherapy (cognitive behavioral therapy, and interpersonal psychotherapy) and exercise therapy for mild to moderate depression [25] but perhaps not really applicable in severe dementia patients.

Polypharmacy was associated with lower HRQoL on the caregiver score of the QoL-AD, and tended to be linked to poorer HRQoL on the patient evaluation. Analysis of the individual items of the QoL-AD showed that polypharmacy was significantly associated with lower scores on the physical health item, and this item alone was common to both the patient and caregiver reports. This relation is logical, since the need for many medications generally stems from the presence of multiple comorbidities, and the patient’s perception of their own health is heavily influenced by the presence of such comorbid diseases. Similarly, a caregiver who sees their loved one suffering from several comorbid diseases is highly likely to rate the patient’s HRQoL more negatively. In our study, comorbidities were assessed using the Charlson index, which was not found to be associated with HRQoL. This may be due to the limited ability of the Charlson index to record the whole spectrum of diseases in older patients [26].

Anxiety showed a tendency to be associated with lower HRQoL on the patient evaluation, independently of the presence of depression but was not associated with HRQoL in the caregiver evaluation. A relation between anxiety and HRQoL has previously been reported [7, 18, 27]. Analysis of the individual items of the QoL-AD showed that anxiety was significantly related to lower HRQoL on the mood, memory and self-esteem items. The presence of depression also influenced the mood and memory scores in the patient reports. As regards the caregiver report, anxiety was not found to be associated with any items, but depression significantly influenced the same items, namely mood, memory and self-esteem. Symptoms of anxiety may simply be confused with depressive symptoms by caregivers, although they may appear before depression, or even be harbingers of impending depression. Anxiety often goes hand in hand with sleep disorders, and sometimes irritability or even anxious agitation. It is therefore not a negligible symptom, especially given than it can be successfully treated with medication and also by psychological methods that help the patient to express and externalize their worries.

In our study, existence of caregiver burden was found to be significantly associated with the caregiver’s score on the QoL-AD. The majority of patients with AD who live at home are cared for by an informal caregiver, usually a member of their family (spouse or child). This caregiver bears the burden of care, and the weight of this burden increases as the disease progresses and requires increasingly complex care. It has been shown that the QoL of caregivers of patients with dementia was lower than that of caregivers for patients with other chonic diseases such as cancer [28]. Moreover, a person’s QoL is known to influence their productivity in the work environment, as well as the rate of absenteeism for sick leave [29]. It is therefore plausible that the QoL of the caregiver of a patient with AD influences the quality of the care that caregiver can provide. Our study shows that caregiver burden influences the caregiver’s perception of almost all the items of the QoL-AD, particularly the items “relationship with family” and “relationship with friends”. For a patient with AD, their caregiver serves as a bridge to maintaining relations with their entourage. The presence of a significant burden renders the caregiver less available to perform this fundamental role. Caregiver burden also influenced the items “ability to do things for fun” and “ability to do chores”. Both these items are subject to the patient’s capacity for initiative, and if the patient has impaired initiative, then the caregiver have an essential role in stimulating the patient to perform activities in both these domains. A caregiver who is exhausted probably prefers to do the housework themselves, rather than accompany the patient, and taking the time to oversee the activities with benevolence and good will. The same is true for leisure activities. A key role of the caregiver is to propose leisure activities to the patient, or at least provide the logistics to enable the patient to participate in such activities, either by taking the patient to community-organized activities or day-centres etc. The mood item was also influenced by the presence of caregiver burden, which could be explained by the fact that AD patients are often highly sensitive to the emotions expressed by their entourage. If the caregiver is tired or depressed, the patient is likely to be aware of this, and may feel guilty for causing this state of stress, insofar as the patient is conscious of their own disease, as guilt is a leading symptom in depression. The burden of the caregiver is little perceived by the patient with Alzheimer’s disease, especially when the disease progresses, mainly due to the fact that it is little expressed by the caregiver towards his patient. The burden of this caregiver is an embarrassment that he/she feels internally and that he/she communicates on solicitation to his/her entourage and/or other caregivers who accompany him/her in the care of the patient. The burden of the caregiver acts indirectly on the patient because it modifies the relationship of spontaneity and satisfaction in exchanges, the patient often remaining in the capacity to perceive it in the person who helps. This relational exchange, largely emotional, is disturbed by the altered perception of the emotions that exist in AD. It is for these reasons that the burden, even if it is not perceived directly by the patient, has an impact on the patient and the psycho-behavioral alterations that he may present. Accordingly, evaluation of caregiver burden is of paramount importance in the management of patients with AD, especially since caregiver burden can be prevented by initiating formal help structures (such as home nurses, home help for housework, delivery of meals, day-care centre attendance for the patient etc).

Other studies have previously investigated the factors associated with patient and caregiver evaluations of QoL-AD. Some authors found discrepancies in the factors associated with QoL between the patient’s evaluation and the caregiver’s evaluation. Certain authors reported that the caregiver’s score was influenced by behavioural disorders [27, 30]. In comparison to our study, the patient population was difference, because the other studies included patients living in long-term residential care facilities, and the caregivers who evaluated were the healthcare personnel of the nursing home and not the patient’s informal caregiver as in our study. In the study by Chan et al., the authors reported that the severity of AD was associated with the patient’s evaluation of their own QoL [23]. Chan’s study included patients with all stages of AD, and MMSE scores ranging from 6 to 28. Conversely, our study included only patients corresponding to the population in which the QoL-AD instrument was validated, namely patients with mild to moderate AD.

Data in the literature show a discrepancy between the evaluation of QoL by the patient, and the evaluation of that patient’s QoL by their caregiver [4–8]. In our study, depression and polypharmacy were found to be associated with the patient score on the QoL-AD, but also with the caregiver score. The existence of common determinants between these two scores pleads in favour of the ability of the caregiver to assess the parameters influencing the patient’s QoL. In the context of progressive disease such as AD, this finding is of definite clinical interest. Indeed, when the patient’s disease progresses such that the patient can no longer complete QoL evaluations, the caregiver becomes the only reliable source of HRQoL evaluation for the patient. Despite the discrepancies between HRQoL as assessed by patients with AD and HRQoL as assessed by their caregiver, the caregiver’s evaluation can nonetheless be used to guide management when the patient can no longer self-assess, with a view to maintaining the patient’s well-being, since the same factors are associated with both the caregiver’s evaluation and the patient’s own evaluation.

Limitations of this study include the non-inclusion of patients with severe AD. Our results show that the factors influencing QoL in patients with mild to moderate stage AD are the same, regardless of the method used to complete the instrument (self-report or administered). It seems important to validate these findings in patients with severe AD, since it is in this population that administration by another person has the greatest potential utility.

## Conclusions

Our study identified two factors that influenced HRQoL in patients with AD, both by patient self-report and on the caregiver report, namely the presence of depression and polypharmacy. Thus, when the patient can no longer complete QoL evaluations, and despite the discrepancies between HRQoL as assessed by patients with AD and HRQoL as assessed by their caregiver, the caregiver’s assessment may be used to guide patient management, with a view to improving the patient’s quality of life.

## Abbreviations

AD: Alzheimer’s Disease
ADL: Katz’s Activities of Daily Living
BMI: Body mass index
HRQoL: Health-related quality of life
IADL: Lawton’s Instrumental Activities of Daily Living
MMSE: Mini Mental State Examination
NPI: Neuro-Psychiatric-Inventory
QoL-AD: Quality of Life in Alzheimer’s Disease instrument

## References

[1] Weyerer S, Schaufele M. The assessment of quality of life in dementia. Int Psychogeriatr. 2003;15(3):213–8. 14756157

[2] Novella JL, Dhaussy G, Wolak A, Morrone I, Drame M, Blanchard F, Jolly D (2012). Quality of life in dementia: state of the knowledge. Geriatrie et psychologie neuropsychiatrie du vieillissement.

[3] Logsdon RG, Gibbons LE, McCurry SM, Teri L (2002). Assessing quality of life in older adults with cognitive impairment. Psychosom Med.

[4] Jacob L, Han JW, Kim TH, Park JH, Lee SB, Lee JJ, Ryu SH, Kim SK, Yoon JC, Jhoo JH (2017). How different are quality of life ratings for people with dementia reported by their family caregivers from those reported by the patients themselves?. J Alzheimers Dis.

[5] Andrieu S, Coley N, Rolland Y, Cantet C, Arnaud C, Guyonnet S, Nourhashemi F, Grand A, Vellas B, Guio D, Pemille D (2016). Assessing Alzheimer’s disease patients’ quality of life: discrepancies between patient and caregiver perspectives. Alzheimers Dement.

[6] Beer C, Flicker L, Horner B, Bretland N, Scherer S, Lautenschlager NT, Schaper F, Almeida OP (2010). Factors associated with self and informant ratings of the quality of life of people with dementia living in care facilities: a cross sectional study. PLoS One.

[7] Orgeta V, Orrell M, Hounsome B, Woods B, team R (2015). Self and carer perspectives of quality of life in dementia using the QoL-AD. Int J Geriatr Psychiatry.

[8] Zhao H, Novella JL, Drame M, Mahmoudi R, Barbe C, di Pollina L, Aquino JP, Pfitzenmeyer P, Rouaud O, George MY (2012). Factors associated with caregivers’ underestimation of quality of life in patients with Alzheimer’s disease. Dement Geriatr Cogn Disord.

[9] APA. Diagnostic and statistical manual of mental disorders: Dsm-iv. Washington: American Psychiatric Association; 1994;143–147.

[10] McKhann G, Drachman D, Folstein M, Katzman R, Price D, Stadlan EM (1984). Clinical diagnosis of Alzheimer’s disease: report of the NINCDS-ADRDA work group under the auspices of Department of Health and Human Services Task Force on Alzheimer’s disease. Neurology.

[11] Wolak A, Novella JL, Drame M, Guillemin F, Di Pollina L, Ankri J, Aquino JP, Morrone I, Blanchard F, Jolly D (2009). Transcultural adaptation and psychometric validation of a French-language version of the QoL-AD. Aging Ment Health.

[12] Katz S, Ford AB, Moskowitz RW, Jackson BA, Jaffe MW (1963). Studies of illness in the aged. The index of Adl: a standardized measure of biological and psychosocial function. Jama.

[13] Barberger-Gateau P, Commenges D, Gagnon M, Letenneur L, Sauvel C, Dartigues JF (1992). Instrumental activities of daily living as a screening tool for cognitive impairment and dementia in elderly community dwellers. J Am Geriatr Soc.

[14] Wimo A, Jonsson L, Zbrozek A (2010). The resource utilization in dementia (RUD) instrument is valid for assessing informal care time in community-living patients with dementia. J Nutr Health Aging.

[15] Zarit SH, Reever KE, Bach-Peterson J (1980). Relatives of the impaired elderly: correlates of feelings of burden. Gerontologist.

[16] Folstein MF, Folstein SE, McHugh PR (1975). “Mini-mental state”. A practical method for grading the cognitive state of patients for the clinician. J Psychiatr Res.

[17] Sundararajan V, Henderson T, Perry C, Muggivan A, Quan H, Ghali WA (2004). New ICD-10 version of the Charlson comorbidity index predicted in-hospital mortality. J Clin Epidemiol.

[18] Barbe C, Morrone I, Wolak-Thierry A, Drame M, Jolly D, Novella JL, Mahmoudi R. Impact of functional alterations on quality of life in patients with Alzheimer's disease. Aging Ment Health. 2017;21(5):571–576. 10.1080/13607863.2015.1132674 26745259

[19] HAS. Stratégie de prise en charge en cas de dénutrition protéino-énergétique chez la personne agée: Haute Autorité de Santé; 2007. https://www.has-sante.fr/portail/upload/docs/application/pdf/synthese_denutrition_personnes_agees.pdf. Accessed 6 July 2018.

[20] Cummings JL, Mega M, Gray K, Rosenberg-Thompson S, Carusi DA, Gornbein J. The Neuropsychiatric inventory: comprehensive assessment of psychopathology in dementia. Neurology. 1994;44(12):2308–14. 10.1212/wnl.44.12.2308 7991117

[21] Alexopoulos GS, Abrams RC, Young RC, Shamoian CA (1988). Cornell scale for depression in dementia. Biol Psychiatry.

[22] Winter Y, Korchounov A, Zhukova TV, Bertschi NE (2011). Depression in elderly patients with Alzheimer dementia or vascular dementia and its influence on their quality of life. J Neurosci Rural Pract.

[23] Chan IW, Chu LW, Lee PW, Li SW, Yu KK (2011). Effects of cognitive function and depressive mood on the quality of life in Chinese Alzheimer's disease patients in Hong Kong. Geriatr Gerontol Int.

[24] Nikmat AW, Hawthorne G, Al-Mashoor SH (2015). The comparison of quality of life among people with mild dementia in nursing home and home care--a preliminary report. Dementia.

[25] Kok RM, Reynolds CF (2017). Management of Depression in older adults: a review. JAMA.

[26] Harboun M, Ankri J (2001). Comorbidity indexes: review of the literature and application to studies of elderly population. Rev Epidemiol Sante Publique.

[27] Hoe J, Hancock G, Livingston G, Orrell M (2006). Quality of life of people with dementia in residential care homes. Br J Psychiatry.

[28] Moraes SR, Silva LS (2009). An evaluation of the burden of Alzheimer patients on family caregivers. Cad Saude Publica.

[29] Bolge SC, Doan JF, Kannan H, Baran RW (2009). Association of insomnia with quality of life, work productivity, and activity impairment. Qual Life Res.

[30] Nakanishi K, Hanihara T, Mutai H, Nakaaki S (2011). Evaluating the quality of life of people with dementia in residential care facilities. Dement Geriatr Cogn Disord.

